# Intrathecal Administration of Autologous CD34 Positive Cells in Patients with Past Cerebral Infarction: A Safety Study

**DOI:** 10.1155/2013/128591

**Published:** 2013-09-25

**Authors:** Liming Wang, Haijie Ji, Ming Li, Jianjun Zhou, Wen Bai, Zhanqiang Zhong, Na Li, Delin Zhu, Zijia Zhang, Yongjun Liu, Mingyuan Wu

**Affiliations:** ^1^Cell Therapy Center, 323 Hospital of Chinese People's Liberation Army, Xi'an 710054, China; ^2^Alliancells Institute of Stem Cells and Translational Regenerative Medicine & Alliancells Bioscience Co., Ltd., Tianjin 300381, China; ^3^Zhongyuan Union Stem Cell Bioengineering Co., Ltd., Tianjin 300050, China; ^4^Beijing Alliancells-PuRui Bioscience Co., Ltd., Beijing 100052, China; ^5^Department of Cell Biology, Oklahoma State University, Tulsa, OK 74106, USA; ^6^Harold Hamm Diabetes Center and Section of Endocrinology and Diabetes in the Department of Internal Medicine, Department of Physiology, University of Oklahoma Health Sciences Center, Oklahoma City, OK 73104, USA

## Abstract

Regenerative strategies in treatment of stroke have great potential. The goal of the current study was to investigate safety of intrathecal administration of autologous CD34 positive cells in treatment of patients with poststroke. A total of eight male patients with a history of stroke were enrolled. The patients were treated subcutaneously with 5 **μ**g/kg body weight rhG-CSF for 5 consecutive days, and then leukapheresis was performed to concentrate cells for CD34 positive immunoselection. All patients underwent intrathecal administration of CD34 positive cells via lumbar puncture. The primary outcome was safety evaluation for 12-month followup. In addition, behavioral function was evaluated with NIH stroke scale and Barthel index 1, 6, and 12 months after the last treatment, respectively. There were no major adverse events, and abnormal changes of blood tests during the whole treatment process included intrathecal administration and 12-month followup. The main message from the current study was that administration of G-CSF-mobilized autologous CD34 positive cells in patients with poststroke was safe. Future studies with larger population and control group are needed to confirm the safety and investigate the efficacy.

## 1. Introduction

Cerebrovascular disorder, mainly including ischemic and hemorrhagic stroke, is the third cause of death after cardiovascular disease and cancer [1] and the leading cause of long-term disability in adults in China [2]. Although stroke mortality has declined in the past decades, its prevalence and morbidity increased dramatically because of enhanced stroke survival [3]. About 40% of stroke survivors have certain degree of impairment and 15–30% are severely disabled [4]. Therefore, every effort to prevent and treat stroke is urgently warranted.

Up to date, many targets within the cascade of neuronal death have been identified. However, neuroprotective strategies in treating poststroke with sequela are controversial [5]. Since neural progenitor and stem cells were discovered in the adult brain for the first time in 1992 [6], different experimental stroke models indicated that neural progenitor and stem cells with activities of migration and differentiation are activated by hypoxia in brain [7, 8]. Increasing evidence from basic studies indicates that cord blood mononuclear cells, bone marrow mononuclear cells, and bone marrow stromal cells can survive in postischemia tissue and reduce neuronal damage when transplanted into rodents subjected to cerebral infarction [9–12].

Bone marrow-derived CD34 positive cells have previously been used to reconstitute the hematopoietic system after radiation or chemotherapy. Recently, CD34 positive cells have been widely reported to induce therapeutic angiogenesis in animal models of myocardial, peripheral, and cerebral ischemia [13–15]. Circulating bone marrow-derived immature cells, including CD34 positive cells, have been implicated in homeostasis of the cerebral microvasculature [16]. In addition, the level of circulating CD34 positive cells was decreased in patients with vascular-type cognitive impairment [17]. Recently, the positive correlation between the number of circulating CD34 positive cells and patients with acute stroke stress has been demonstrated, which suggests that increased numbers of circulating CD34 positive cells might benefit neurologic function recovery [18]. Although previous clinical data have demonstrated a positive benefit in patients with stroke [19], there are no clinical reports on autologous CD34 positive precursor cells in the treatment of poststroke.

In our ongoing clinical study, we designed a protocol to determine the safety and potential efficacy of G-CSF mobilization of bone marrow-derived CD34 positive cells in the treatment of patients with a history of stroke. In the current study, we focused on the investigation of the safety of CD34 positive cell administration.

## 2. Material and Methods

### 2.1. Clinical Study Design

This was a prospective and observational study. The study was approved by the Institutional Review Boards of 323 Hospital of PLA (Xi'an, China). The study was conducted according to the principles of the Declaration of Helsinki. All study participants provided written informed consent. The procedure of our ongoing clinical study was under STEPS criteria [20, 21].

### 2.2. Patients

Eight male patients with a history of stroke and left sequela were the target population in this study. The inclusion and exclusion criteria are listed in detail in Table 1.

### 2.3. G-CSF, CD34 Positive Cell Harvesting and Immunoselection

All patients were treated subcutaneously with 5 *μ*g/kg body weight G-CSF for five consecutive days. Leukapheresis was performed using a Cobe Spectra cell separator (Cobe Bct, Lakewood, CO). Blood pressure and heart rate were monitored, and a physician was available for any emergencies during the performance. CD34 positive cells were extracted from leukapheresis products by immunoselection using an automated cell separation system (Miltenyi Biotec, Bergisch Gladbach, Germany) based on the mechanism of selectivity of a monoclonal CD34 antibody conjugated to a magnetic particle [22, 23]. For purity analysis of immunoselected CD34 positive cells, cells were evaluated by flow cytometry using CD34-PE-conjugated monoclonal antibody. Then the immunoselected CD34 positive cell components were divided into multiunits stored in liquid phase nitrogen at −196°C and will be infused later. The purity of CD34 positive cells was in the range of 85–90%. 0.8–3.3 × 10^7^ (varying from different patients) in 2 mL medium was used per time. The cells from the Good Manufacturing Practice (GMP) workshop were ensured to have been fully screened against potential pathogens and meet the eligible criteria for clinical use.

### 2.4. Cell Administration

All subjects were hospitalized during the administration of CD34 positive cells. Patients' X-rays, computed tomography scans, and magnetic resonance images of spine were reviewed prior to administration. Intrathecal injection by lumbar puncture was repeated four or five times depending on the patient's condition. Treatments were separated by one-week interval. Patients were given local anesthesia, and lumbar puncture was performed in the lateral decubitus position, prepped, and draped in sterile fashion, and the needle was placed in the lumbar cistern. 2 mL of cerebrospinal fluid was removed and replaced by 2 mL of immunoselected CD34 positive cell suspension.

### 2.5. Safety Evaluation and Followup

Clinical chemistry monitoring, including tests of blood count, liver function, renal function, lipids, and glucose, was performed before, 1 day, and 7 days after cell treatment, respectively. The treatment patients were also under rehabilitation training and followed up for 12 months. Any adverse events that occurred in the treatment were documented. In addition, NIH stroke scale and Barthel index, which are widely used in evaluation of functional disability, were documented at baseline and at 1st, 6th, and 12th months in the 12-month followup.

### 2.6. Statistical Analysis

Parameters measured before and after treatment were compared using a Student's *t*-test. A *P* value of < 0.05 was considered significant.

## 3. Results

### 3.1. Basic Information

As shown in Table 2, 8 male patients (age: 22–58 yrs, median: 42 yrs) with a history of stroke (range: 1–7 yrs, median: 2 yrs) were enrolled. G-CSF and leukapheresis were well tolerated in all patients during the clinical treatment course. A mean of 1.8 × 10^7^ of CD34 positive cells (range 0.8–3.3) was collected by immunoselection. Flow cytometry analysis showed that CD34 positive cells in the immunoselected fraction were above 85%. The patients received four or five times cell transplantation during the whole course.

### 3.2. Safety Evaluation

No allergic or immunological reactions were noted at the time of injection or while under observation. No chest pain and abnormal level of cardiac enzymes were observed. In addition, there is no statistical difference of blood tests (Tables 3 and 4) before treatment, 1 day, and 7 days after treatment. Therefore, the above data indicated that cell infusion into the subarachnoid space was a safe procedure in patients with poststroke.

### 3.3. Efficacy Evaluation

As shown in Table 5, we observed that the daily activities had a gradual improvement tendency in the 12-month followup. Mean of NIH stroke scale and BI scores shifted from 7.5 and 43.8 at baseline to 4.4 and 64.4 in 12 months later, respectively. There was no significant difference one month after the treatment compared to baseline. Improvement was observed 6 and 12 months after the last treatment. The improvements of muscle tone, rigidity, and motor power were observed in all patients. However, we must emphasize that patients with stroke do gradually improve their functions, and here it is not possible to define whether the therapy itself or the natural history of the disease is responsible for the improvements noted.

## 4. Discussion

In the current study, we observed that G-CSF administration, leukapheresis, and intrathecal administration of CD34 positive cells were safe and well tolerated in a group of patients with past cerebral infarction with 12-month followup.

G-CSF has been used extensively in the last decades to mobilize CD34 positive hematopoietic stem cells in neutropenic patients and for reconstitution of bone marrow [24, 25]. Recently, G-CSF as a promising drug candidate can reduce neuroinflammation and potentiate both neurogenesis and angiogenesis after ischemic stroke by promoting bone marrow cell migration into the ischemic brain [26, 27]. Many years of clinical experience with G-CSF have shown the safety of this agent in the general population [28]. Increasing clinical studies indicated mobilization of CD34 positive precursor cells in patients with acute stroke by G-CSF is safe and effective [29–31]. In the current study, patients with past cerebral infarction were treated by G-CSF; subsequently, leukapheresis was performed for CD34 positive cells collection by immunoselection. There were no emergencies, and adverse events, such as hyperviscosity syndromes, occurred in the whole process.

Previously, it has been reported that intrathecal administration was safe with a variety of stem cells including adipose tissue-derived mesenchymal stem cells, embryonic stem cell-derived hematopoietic stem cells, and autologous bone marrow-derived hematopoietic stem cells in the treatment of neurological disorders [32, 33]. In this study, intrathecal injection of the CD34 positive cells into subarachnoid space was performed, thereby migrating more efficiently into the injured area related to stroke through cerebrospinal fluid compared to the intravenous route. What is more, there were no side effects including headache, low-grade fever, and meningism and vomit occurring in eight patients.

CD34 positive cells comprise a population enriched with endothelial progenitor cells whose contribution to neovasculature includes both direct participation in forming the neovessel and regulatory roles and the sources of growth/angiogenesis factors including VEGF, HGF, and IGF-1 [34], contributing to the maintenance of the microvasculature. The positive correlation between levels of circulating CD34 positive cells and neovascularization in patients with postcerebral ischemia was observed [35]. In this study, given the variance of age, transplantation cell numbers, and stroke history, the discrepant response to the treatment is apprehensible. The current data showed a trend that CD34 positive cells might achieve clinical benefits in the treatment of patients with poststroke. However, improvements do occur naturally in patients with stroke, and it is not possible to define whether the therapy itself or the natural history of the disease was responsible for the improvements noted.

In conclusion, this study indicates that intrathecal administration of CD34 positive cells in patients with past stroke is a relatively safe and simple procedure with no long-term adverse effects. However, further larger clinical studies with multiple groups including blinding and placebo controls will be required to confirm our findings in the current study.

## Figures and Tables

**Table 1:** Inclusion and exclusion criteria.

Inclusion criteria	Exclusion criteria
Age ≥18 years to ≤60 years	Systemic malignancy
Patient must comprehend the study protocol	Hematological system disorder (e.g., myeloproliferative disorder)
Patient must be compliant	Thrombocyte function disorders
Patient provided written informed consent	Metabolic syndrome with inadequate treatment parameters, for instance, excessive hypertension, hyperlipemia, and hyperglycemia
	Known deficit in hemostasis
	Serious coronary heart disease
	Sickle cell anemia
	Allergy against G-CSF
	Pregnancy
	Any other serious disease, for instance, severe psychiatric disorder (major depression, schizophrenic psychosis, and addiction), severe cardiac disorder with hemodynamic relevance and positive HIV serology
	Epilepsy Hydrocephaly

**Table 2:** The basic information of eight patients.

Patient	Gender	Age (yrs)	Stroke age (yrs)	CD34 positive cells (×10^7^)	Number of times
1	Male	58	4	1	4
2	Male	43	7	0.8	4
3	Male	41	1	2.7	5
4	Male	44	3	1.4	4
5	Male	22	1	1.2	4
6	Male	40	2	3.3	4
7	Male	47	2	0.9	4
8	Male	37	1	3.1	5

**Table 3:** Changes of RBC, WBC, PLT, and HB in blood before and after treatment (*n* = 8, mean ± SD).

Time points	RBC (×10^12^/L)	WBC (×10^9^/L)	PLT (×10^9^/L)	HB (g/L)
Baseline	4.7 ± 0.6	8.9 ± 1.3	204.4 ± 89.0	127.5 ± 11.6
1 day	3.9 ± 0.4	7.5 ± 1.6	217.1 ± 70.8	122.6 ± 17.3
7 day	4.1 ± 0.8	7.9 ± 1.8	207.3 ± 97.8	121.7 ± 16.1

RBC: red blood cells; WBC: white blood cells; PLT: platelet; HB: hemoglobin.

**Table 4:** The blood biochemical analysis before and after treatment (*n* = 8, mean ± SD).

Item (unit)	Baseline	1 day	7 day
TP (g/L)	64.4 ± 6.1	66.4 ± 5.9	65.3 ± 5.6
ALB (g/L)	39.4 ± 4.6	41.4 ± 3.8	41.1 ± 3.7
GLB (g/L)	25.6 ± 4.5	26.4 ± 4.9	24.3 ± 5.0
TBIL (*μ*mol/L)	7.8 ± 1.4	8.6 ± 1.1	6.9 ± 2.8
DBIL (*μ*mol/L)	3.1 ± 0.8	3.3 ± 0.6	2.7 ± 1.0
ALT (g/L)	25.2 ± 6.9	37.2 ± 4.5	27.3 ± 13.4
AST (*μ*mol/L)	17.5 ± 3.3	27.9 ± 5.2	21.8 ± 9.1
UA (*μ*mol/L)	240.7 ± 38.5	256.9 ± 71.5	239.5 ± 53.9
Cr (*μ*mol/L)	50.5 ± 12.1	48.6 ± 11.5	45.6 ± 11.2
BUN (mmol/L)	5.3 ± 1.4	5.6 ± 1.7	4.1 ± 0.93
CHOL (mmol/L)	4.5 ± 0.61	4.3 ± 0.90	4.1 ± 0.88
TG (mmol/L)	1.1 ± 0.37	1.3 ± 0.46	1.2 ± 0.82
GLU (mmol/L)	4.8 ± 0.74	5.1 ± 0.38	4.9 ± 0.58

TP: total protein; ALB: albumin; GLB: globulin; TBIL: total bilirubin; DBIL: direct bilirubin; ALT: alanine aminotransferase; AST: aspartate aminotransferase; UA: uric acid; Cr: creatinine; BUN: blood urea nitrogen; CHOL: cholesterol; TG: triglyceride; GLU: glucose.

**Table 5:** The comparison of NIHSS score and BI score before and followed up for 1, 6, and 12 months after the last cell transplantation.

Patient	NIHSS score				BI score			
	Baseline	1	6	12	Baseline	1	6	12
1	8	8	7	6	35	35	40	65
2	3	3	2	1	40	45	55	80
3	17	16	11	7	0	0	10	25
4	3	3	3	2	65	65	65	80
5	4	4	3	1	70	70	75	80
6	3	3	3	2	70	70	70	80
7	2	2	1	0	70	70	80	95
8	20	20	17	16	0	5	5	10
Mean	7.5	7.4	5.9	4.4	43.8	45	50	64.4
*P* value	—	>0.05	>0.05	<0.05	—	>0.05	<0.05	<0.01

The NIHSS score and BI score of 1, 6, and 12 month were compared to the corresponding baseline, respectively.
